# *Galleria mellonella* possesses the essential nutritional needs to host the fastidious Huanglongbing bacterial pathogen ‘*Candidatus* Liberibacter asiaticus’

**DOI:** 10.1038/s42003-025-08802-5

**Published:** 2025-09-30

**Authors:** Nabil Killiny, Yasser Nehela, Faraj Hijaz, Mahnaz Rashidi, Shelley E. Jones

**Affiliations:** 1 Department of Plant Pathology, Citrus Research and Education Center, IFAS, University of Florida, 700 Experiment Station Road, Lake Alfred, FL 33850 USA; 2 Department of Agricultural Botany, Faculty of Agriculture, Tanta University, Tanta, 31527 Egypt

**Keywords:** Gas chromatography, Parasite host response

## Abstract

Citrus greening disease, caused by *‘Candidatus* Liberibacter asiaticus’, severely impacts citrus production worldwide. The development of sustainable control strategies for this disease is restricted by the unavailability of the bacterium in pure culture. Herein, the metabolic profile of the waxworm larvae, *Galleria mellonella*, was compared to that of *Diaphorina citri*, the vector of *‘Ca*. L. asiaticus’. Our findings showed that *G. mellonella* larvae possess the nutritional needs to host ‘*Ca*. L. asiaticus’, supporting its short-term persistence, and responds to infection with a visible immune reaction by producing melanin upon bacterial invasion. The inoculated larvae exhibit detectable bacterial titers for up to four days when inoculated with infected citrus phloem sap or *D. citri* haemolymph, after which bacterial titers decline, and infected larvae show reduced survival compared to mock-treated and ‘*Ca*. L. asiaticus’-free controls. Metabolic profiling of *G. mellonella*, *D. citri*, and honeybees (*Apis mellifera*) reveals distinct chemical compositions in their haemolymph. *G. mellonella* contains higher levels of amino acids, organic acids, nucleotides, and sugar-nucleotides, providing essential nutrients for *‘Ca*. L. asiaticus’, while *D. citri* is enriched in monosaccharides and sugar-alcohols. Citric acid was detected exclusively in the haemolymph of *G. mellonella*. These findings suggest *G. mellonella* as a convenient model that can transiently host *‘Ca*. L. asiaticus’ for short-term use, which would facilitate high-throughput screening of antimicrobial compounds against ‘*Ca*. L. asiaticus’, as well as exploring host-pathogen interactions. This model could accelerate the development of effective treatments against citrus greening and inform broader strategies for managing vector-borne plant diseases.

## Abstract (web summary)

*Galleria mellonella* possesses the nutritional needs to host the fastidious phytopathogenic bacterium ‘*Ca*. L. asiaticus’, supporting its short-term persistence and providing a model for rapid screening of antimicrobial treatments against Huanglongbing.

## Introduction

Huanglongbing (HLB; aka citrus greening disease) is one of the most devastating diseases affecting citriculture worldwide^1^. The disease is caused by the phloem-restricted fastidious bacterium *‘Candidatus* Liberibacter asiaticus*’* which is transmitted by the Asian citrus psyllid, *Diaphorina citri*^1^. Since its emergence in Florida in 2005, HLB has caused significant economic losses, threatening the sustainability of the citrus industry. ‘*Ca*. L. asiaticus’ infects the entire plant from shoot to root and eventually causes loss of root mass^2^, chlorotic leaves, and premature fruit drop^3^; all of which eventually lead to reduced fruit yield and often tree death. Additionally, infection with ‘*Ca*. L. asiaticus’ triggers several metabolic imbalances^4^, phytohormonal changes^5^, accumulation of starch^6^, and suppresses both localized and systemic innate immunity and lipopolysaccharide-mediated defense signaling of infected citrus plants^7^. Unfortunately, no sustainable, effective cure has been developed against HLB yet, mainly because of the inability to culture ‘*Ca*. L. asiaticus’ in vitro.

Despite decades of research, ‘*Ca*. L. asiaticus’ remains not available in culture under axenic conditions. Early trials involving traditional bacterial media failed to support ‘*Ca*. L. asiaticus’ growth under standard laboratory conditions. This might be mainly due to the highly reduced genome of ‘*Ca*. L. asiaticus’^8^, which suggests a strong dependence on host-derived nutrients. The genome of ‘*Ca*. L. asiaticus’ lacks several essential genes for key biosynthetic pathways for amino acids, vitamins, and cofactors, as well as carbohydrate metabolism^8^. Previous studies have attempted the co-cultivation of ‘*Ca*. L. asiaticus’ with some actinobacteria^9^ or the addition of vein extract^10^ or juice^11,12^ to the culture medium. However, these attempts only prolonged the viability of ‘*Ca*. L. asiaticus’ for very few single-colony transfers before it rapidly declined^10,11^. Recent trials focused on the development of chemically defined media^13^ or using host-free microbial culture^14–16^. Nevertheless, these cultures failed to achieve sustained proliferation or reproducibility. Moreover, it was demonstrated that the striped mealybug (*Ferrisia virgata*) can acquire and retain *‘Ca*. L. asiaticus’, with bacterial titers increasing over time^17^. However, *‘Ca*. L. asiaticus’ populations in mealybugs differ genetically, particularly in prophage profiles, from those in psyllids, and were unable to cause disease in plants, suggesting vector-specific genotype selection influences pathogen behavior^17^.

Regardless of occasional reports of transient growth, a reproducible and reliable culture medium has yet to be established, highlighting the challenges associated with the pathogen’s nutritional needs and host-specificity. The lack of a suitable culture system has been a barrier to understanding the biology and physiology of ‘*Ca*. L. asiaticus’, fulfilling Koch’s postulates, identifying potential antibacterial targets, and developing sustainable disease management strategies. The culturing difficulty of *‘Ca*. L. asiaticus’ has driven researchers to depend on surrogate systems, such as *Liberibacter crescens*, an axenically culturable relative of *‘Ca*. L. asiaticus’, for functional analysis of *‘Ca*. L. asiaticus’ genes^18–20^. Although *L. crescens* has provided valuable insights into the biology, pathogenesis, and physiological dependence of *‘Ca*. L. asiaticus’ on the host cell, it does not fully replicate the distinctive characteristics and nutritional needs of *‘Ca*. L. asiaticus’. Alternatively, researchers have relied heavily on infected plant tissues^21^ or psyllid vectors^22^ to indirectly investigate the biology of *‘Ca*. L. asiaticus’. Nevertheless, these methods are labor-intensive, technically challenging, and often yield inconsistent results due to the low bacterial titers present in infected hosts. Therefore, there is a desperate need for alternative models that can support the growth and viability of ‘*Ca*. L. asiaticus’ and facilitate its study under controlled conditions.

The larvae of *Galleria mellonella* (greater wax moth), a member of the order Lepidoptera, have emerged as a promising invertebrate model for studying several fungi^23^, yeasts^24^, bacteria^25^, and many others^26^. Moreover, *G. mellonella* has complemented or supplanted the traditional animal models, such as *Drosophila* and mouse models, for studying pathogens^27^ because of its ability to represent the mammalian innate immune system response without significant costs or ethical concerns.

In addition to its reduced innate immune system, *G. mellonella* hemolymph provides a nutrient-rich environment^28^ that mimics some aspects of host tissues, including the presence of ATP, a critical energy source that ‘*Ca*. L. asiaticus’ cannot synthesize independently^29^. Moreover, *G. mellonella* larvae offer numerous advantages over traditional models. They are inexpensive to maintain, ethically favorable compared to vertebrate models, and capable of surviving at a wide range of temperatures (25–37°C), which aligns with the optimal temperature range for ‘*Ca*. L. asiaticus’ growth^30^. Additionally, *G. mellonella* larvae are relatively large, which makes inoculation, handling, and rearing easier compared to *D. citri*. Collectively, this makes *G. mellonella* a potentially suitable host for supporting the survival of ‘*Ca*. L. asiaticus’. However, to the best of our knowledge, there are no previous reports of the persistence of fastidious bacterial phytopathogens in the *G. mellonella* infectious host^31^.

‘*Ca*. L. asiaticus’ has a dual lifestyle, multiplying in both citrus phloem sap and its insect vector. Within *D. citri*, the ‘*Ca*. L. asiaticus’ bacterium is propagative and circulative within the insect’s body and multiplies within the haemolymph. We hypothesize that because the ‘*Ca*. L. asiaticus’ bacteria grow in the haemolymph of its vector; it may also grow, or at least persist, in the haemolymph of *G. mellonella*. To test this hypothesis, the haemolymph chemical composition of *D. citri*^32^, as well as *G. mellonella*^31^, were previously studied individually. Similarities in the haemolymph composition of both systems lead us to hypothesize that the much larger and more easily reared *G. mellonella* might be useful for sustaining ‘*Ca*. L. asiaticus’ persistence and viability. However, more chemical analyses are needed to identify compounds that are missing in *G. mellonella* haemolymph compared to *D. citri* haemolymph.

Developing an alternative host system that can support the essential nutritional needs of ‘*Ca*. L. asiaticus’, might bridge critical gaps between surrogate studies using *L. crescens* and field-based research involving citrus plants and psyllid vectors. Building on an earlier study that reported ‘*Ca*. L. asiaticus’ persistence and potential genotype selection in mealybugs^17^, our study complements this by demonstrating short-term persistence in a non-psyllid insect via direct inoculation. The current study investigates the potential of *G. mellonella* as a surrogate host for the fastidious Huanglongbing bacterial pathogen, ‘*Ca*. L. asiaticus’ and evaluates its ability to meet the bacterium’s essential nutritional needs via comparative chemical analyses of the haemolymph of *D. citri, G. mellonella*, and the honeybee (*Apis mellifera*), as a non-vector to ‘*Ca*. L. asiaticus’. Furthermore, the effect of ‘*Ca*. L. asiaticus’ infection (using infected phloem sap or *D. citri* haemolymph) on the survival and lifespan of *G. mellonella* larvae was also studied.

## Results

### ***G. mellonella*** larvae produce melanin in response to **‘*Ca****.* L. asiaticus’ invasion

**Fig. 1:**
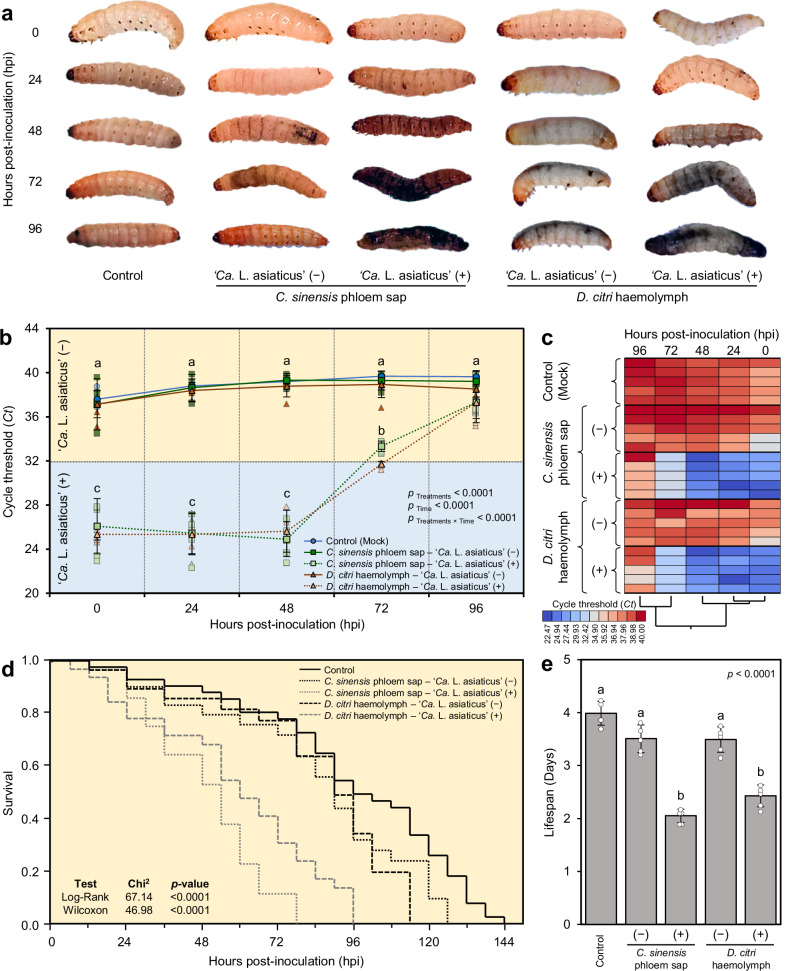
Effect of infection with ‘*Ca*. L. asiaticus’ on the survival of *G. mellonella* larvae. **a** Melanization on *G. mellonella* larvae exposed to infection with ‘*Ca*. L. asiaticus’ via *C. sinensis* phloem sap or *D. citri* haemolymph over 96 h post-inoculation (hpi). Phosphate-buffered saline (0.1 M, pH 7.4) containing 0.1% ascorbic acid (w/v) as an antioxidant (hereinafter referred to as PBS) was used as a negative control. **b** Cycle threshold (*Ct*) of real-time qPCR for the detection of ‘*Ca*. L. asiaticus’ bacterial titer within *G. mellonella* larvae exposed to infection with ‘*Ca*. L. asiaticus’ via *C. sinensis* phloem sap or *D. citri* haemolymph over 96 hpi (*n* = 5). Horizontal thick lines indicate the medians, boxes show the interquartile ranges including 25%−75% of the values, and whiskers reflect the highest and the lowest values of the data. Different letters indicate statistically significant differences among treatments, same letters signify no significant differences among treatments using the Tukey-Kramer honestly significant difference (HSD) test (*p* < 0.05). **c** Hierarchical Cluster Analysis (HCA) and its associated heat map of Cycle threshold (*Ct*) of real-time qPCR for the detection of ‘*Ca*. L. asiaticus’ bacterial titer within *G. mellonella* larvae exposed to infection with ‘*Ca*. L. asiaticus’ via *C. sinensis* phloem sap or *D. citri* haemolymph over 96 hpi. Rows represent the *Ct*, whereas columns represent different time points (0, 24, 48, 72, and 96 hpi). Low Ct levels (higher bacterial titers) are colored gradient blue, whereas high Ct levels (lower bacterial titers) are colored gradient red (see the scale at the bottom left corner of the heat maps). Raw numerical data underlying the graph are provided in Supplementary Data 1. **d** Kaplan-Meier analysis of survival probability of *G. mellonella* larvae exposed to infection with ‘*Ca*. L. asiaticus’ via *C. sinensis* phloem sap or *D. citri* haemolymph over four days post-inoculation (dpi) (*n* = 25). *P-*values and Χ^2^ of log-rank and Wilcoxon tests (presented in the upper right corner of the graph) were used for statistical comparisons among the survival curves. Raw numerical data underlying the graph are provided in Supplementary Data 2. **e** Lifespans associated with the cumulative survival of *G. mellonella* larvae with different ‘*Ca*. L. asiaticus’ infection. Bars and error bars represent means and SDs, respectively. Different letters indicate statistically significant differences among treatments, whereas the same letters signify no significant differences between treatments using Tukey HSD (*p* < 0.05). Raw numerical data underlying the bar chart are provided in Supplementary Data 3.

Over four days post-inoculation (dpi), *G. mellonella* larvae were exposed to infection with ‘*Ca*. L. asiaticus’ via *C. sinensis* phloem sap or *D. citri* haemolymph exhibited progressively noticeable signs of melanization compared to those in the non-infected or those treated with ‘*Ca*. L. asiaticus’-free phloem sap or haemolymph (Fig. 1a). Melanization began with the formation of black or brown spots on the surface of the cuticle of treated larvae at 2 dpi, then gradually spread throughout the larva’s body based on the extent of ‘*Ca*. L. asiaticus’ invasion. It is worth mentioning that the body of *G. mellonella* larvae that was inoculated with ‘*Ca*. L. asiaticus’-infected phloem sap exhibited faster and greater melanization than other treatments, becoming completely brownish or black at 3-4 dpi, indicating a severe infection that eventually results in the death of the larva (Fig. 1a).

### Temporal Dynamics of ‘*Ca*. L. asiaticus’ Persistence in *G. mellonella*

**Fig. 2:**
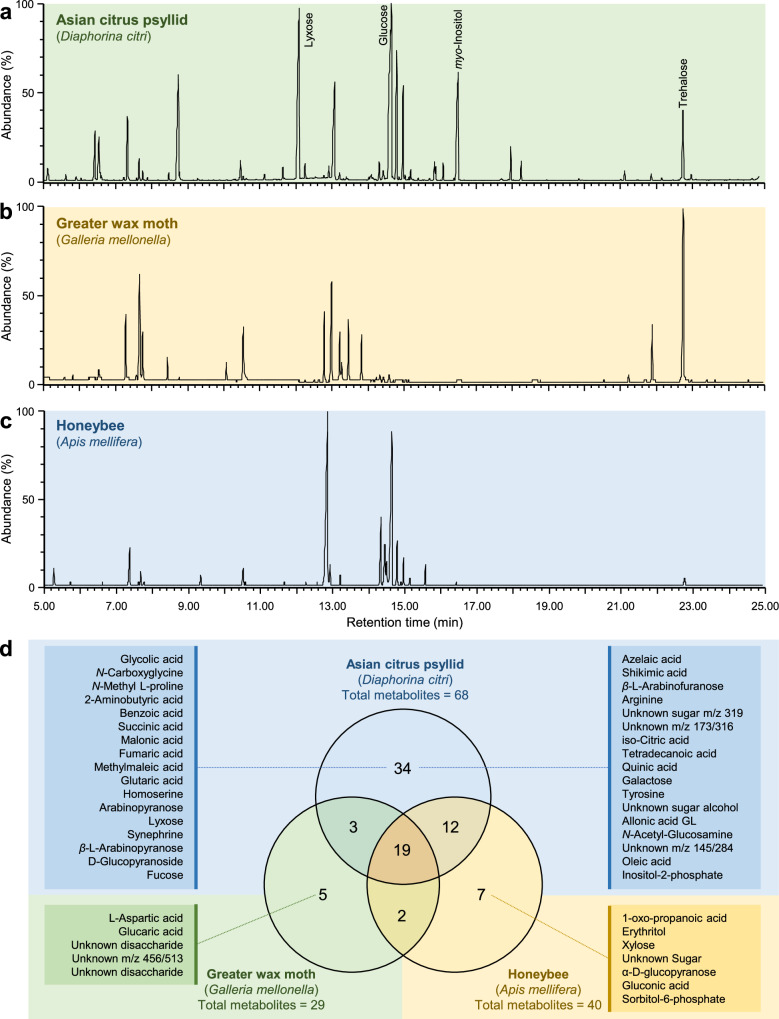
Representative total ion chromatograms (TIC) of haemolymph metabolites after derivatization with trimethylsilyl TMS and analysis using GC-MS running in the full scan mode. **a** Haemolymph of Asian citrus psyllid (*Diaphorina citri*), (**b**) Haemolymph of Greater wax moth (*Galleria mellonella*), (**c**) Haemolymph of Honeybee (*Apis mellifera*). Annotations for the main metabolites are shown on the chromatograms. **d** Venn Diagram of shared metabolites between all three insect species (*n* = 3).

Quantitative real-time PCR (qPCR) analysis revealed distinct temporal patterns in *‘Ca*. L. asiaticus’ titers within *G. mellonella* larvae following inoculation with either *‘Ca*. L. asiaticus’-infected *C. sinensis* phloem sap or *D. citri* haemolymph (Fig. 2b). Larvae exposed to *‘Ca*. L. asiaticus’-negative treatments (mock control, uninfected phloem sap, and uninfected haemolymph) consistently exhibited high cycle threshold (*Ct* > 32) values across all measured time points (0–96 h post-inoculation (hpi)), indicating the absence of bacterial DNA. On the other hand, *G. mellonella* larvae inoculated with *‘Ca*. L. asiaticus’-positive phloem sap or haemolymph displayed significantly lower C_t_ values (<32) at 0, 24, and 48 hpi, consistent with a high bacterial load shortly after inoculation (Fig. 2b). Notably, these low C_t_ values remained stable through 48−72 hpi, suggesting that *‘Ca*. L. asiaticus’ persisted within the larval hemocoel during this early infection window. However, a marked increase in C_t_ values was observed at 72−96 hpi, indicating a progressive decline in bacterial titer.

Hierarchical cluster analysis (HCA) and its corresponding heatmap (Fig. 1c) visually reinforce these trends. HCA demonstrates that *G. mellonella* larvae can support the short-term persistence of ‘*Ca*. L. asiaticus’ for at least 48−72 hpi, which gradually transitioned to red hues at later time points, reflecting reduced pathogen levels. In contrast, ‘*Ca*. L. asiaticus’-negative treatments exhibited higher *Ct* values (>32) throughout the experiment, consistent with the absence of detectable bacterial DNA (Fig. 1c). The observed temporal resolution of bacterial persistence underscores the potential utility of *G. mellonella* as a short-term in vivo system for ‘*Ca*. L. asiaticus’ research.

### **‘***Ca*. L. asiaticus’ invasion reduced the longevity of *G. mellonella* larvae

Cumulative survival over 6 days showed that larvae exposed to ‘*Ca*. L. asiaticus’ infection, particularly with infected phloem sap, had a lower survival probability compared to other treatments (*n* = 25, *χ*^2^ = 67.14 and 46.98 for Log-Rank and Wilcoxon tests, respectively, *P* < .0001 for both; Fig. 1c). Furthermore, the lifespans of *G. mellonella* larvae exposed to infection with ‘*Ca*. L. asiaticus’ via phloem sap or *D. citri* haemolymph was significantly reduced (Fig. 1d). On the other hand, no significant differences were observed in either survival probability or lifespans of *G. mellonella* larvae inoculated with ‘*Ca*. L. asiaticus’-free phloem sap and haemolymph, compared to the mock control (Fig. 1c, d).

### TMS-based derivatization reveals differential haemolymph chemical composition between *D. citri*, *G. mellonella*, and *A. mellifera*

TMS-based non-targeted metabolic profiling of three insect species, *D. citri* (Fig. 2a), G*. mellonella* (Fig. 2b), and *A. mellifera* (Fig. 2c), revealed distinct differences in carbohydrate, sugar alcohols, carboxylic acids, amino acids, amines, and phenols composition, highlighting species-specific metabolic adaptations (Table 1). The metabolomic analysis of three insect species revealed distinct and overlapping metabolic profiles. Generally, 82 metabolites were detected using GC-MS after TMS derivatization, however, only 19 core metabolites were found in common among the three hemolymphs (Table 1 and Fig. 2). The haemolymph of *D. citri* contained the highest number of detected metabolites, totaling 68 compounds, 34 of which were unique (Table 1 and Fig. 2d). On the other hand, the hemolymphs of *G. mellonella* and *A. mellifera* possessed 40 and 29 metabolites, respectively, with seven and five unique compounds (Fig. 2d). Moreover, while 12 metabolites were common between the *D. citri* and *A. mellifera*, only three were shared between the *D. citri* and *G. mellonella* (Fig. 2d). It is worth noting that, after FDR correction, all detected metabolites met the adjusted significance threshold (*q* < 0.05), indicating that the observed differences are statistically significant after accounting for multiple comparisons.

**Table 1:** Hemolymph chemical composition of Asian citrus psyllid (*Diaphorina citri*), greater wax moth (*Galleria mellonella*), and honeybee (*Apis mellifera*) after TMS derivatization using GC-MS (*n* = 3) ^a^

TMS metabolite	RT ^b^	LRI ^c^	Asian citrus psyllid (*Diaphorina citri*)	Greater wax moth (*Galleria mellonella*)	Honeybee (*Apis mellifera*)	*p-*value	FDR-adjusted *p*-value ^d^
Glycolic acid	4.83	1067	Trace	ND ^g^	ND	NC ^h^	NC
_L_-Alanine ^e^	5.14	1087	4.14 ± 0.56	2.04 ± 0.10	2.43 ± 2.13	0.0009	0.0012
Oxalic acid ^f^	5.47	1114	0.92 ± 0.05	ND	0.16 ± 0.17	0.0002	0.0002
*N*-Carboxyglycine	5.61	1129	0.40 ± 0.21	ND	ND	NC	NC
*N*-Methyl L-proline	6.10	1166	0.29 ± 0.14	ND	ND	NC	NC
2-Aminobutyric acid	6.59	1222	0.70 ± 0.40	ND	ND	NC	NC
Valine ^e^	6.62	1225	0.59 ± 0.05	1.91 ± 0.10	0.27 ± 0.13	< 0.0001	0.0036
Benzoic acid	6.97	1255	0.16 ± 0.01	ND	ND	NC	NC
2-Aminopropanol ^f^	7.20	1271	1.30 ± 0.02	ND	0.30 ± 0.07	< 0.0001	0.0018
Phosphoric acid ^e^	7.30	1278	71.62 ± 3.32	10.97 ± 0.40	3.86 ± 2.29	< 0.0001	0.0012
Isoleucine ^e^	7.60	1297	0.22 ± 0.07	1.22 ± 0.06	0.61 ± 0.59	0.0324	0.0353
_L_-Proline ^e^	7.71	1307	6.35 ± 0.72	22.41 ± 1.65	0.91 ± 0.76	< 0.0001	0.0009
Glycine ^e^	7.81	1310	1.46 ± 0.14	7.93 ± 0.39	0.70 ± 0.24	< 0.0001	0.0007
Succinic acid	7.90	1315	0.16 ± 0.01	ND	ND	NC	NC
1-Oxo-propanoic acid	8.10	1338	ND	ND	0.08 ± 0.02	NC	NC
Malonic acid	8.26	1351	0.19 ± 0.01	ND	ND	NC	NC
Fumaric acid	8.35	1358	0.19 ± 0.00	ND	ND	NC	NC
Methylmaleic acid	8.42	1363	0.05 ± 0.01	ND	ND	NC	NC
_L_-Serine ^e^	8.48	1372	1.00 ± 0.06	5.69 ± 0.52	0.35 ± 0.04	< 0.0001	0.0006
_L_-Threonine	8.73	1395	0.84 ± 0.02	0.60 ± 0.01	0.70 ± 0.26	0.2324	0.2390
Glutaric acid	9.20	1421	Trace	ND	ND	NC	NC
*β*-Alanine ^e^	9.35	1447	Trace	0.39 ± 0.01	2.19 ± 1.12	0.0129	0.0150
Homoserine	9.55	1467	Trace	ND	ND	NC	NC
Malic acid ^e^	10.05	1515	0.30 ± 0.02	2.43 ± 0.13	0.09 ± 0.06	< 0.0001	0.0005
Erythritol	10.20	1522	ND	ND	0.19 ± 0.01	NC	NC
_L_-Aspartic acid	10.45	1538	ND	0.44 ± 0.00	ND	NC	NC
Pyroglutamic acid ^e^	10.46	1541	3.73 ± 0.29	8.96 ± 0.39	1.22 ± 1.11	< 0.0001	0.0005
GABA ^f^	10.62	1552	0.70 ± 0.02	ND	0.34 ± 0.13	0.0090	0.0108
Threonic acid ^f^	10.89	1567	0.19 ± 0.02	ND	4.47 ± 0.03	< 0.0001	0.0004
2-Ketoglutaric acid ^f^	11.12	1580	0.43 ± 0.02	1.52 ± 0.02	ND	< 0.0001	0.0004
_L_-Asparagine ^f^	11.40	1600	0.51 ± 0.01	0.68 ± 0.01	ND	< 0.0001	0.0003
Arabinopyranose	11.52	1608	14.38 ± 0.59	ND	ND	NC	NC
Glutamic acid ^e^	11.63	1637	0.19 ± 0.03	0.26 ± 0.00	0.26 ± 0.12	0.4153	0.4153
_L_-Phenylalanine ^f^	11.69	1641	0.05 ± 0.00	0.37 ± 0.01	ND	< 0.0001	0.0003
Xylose	11.98	1679	ND	ND	0.29 ± 0.10	NC	NC
Lyxose	12.30	1711	2.68 ± 0.05	ND	ND	NC	NC
Synephrine	12.37	1724	0.57 ± 0.02	ND	ND	NC	NC
*β*-_L_-Arabinopyranose	12.52	1726	2.41 ± 0.01	ND	ND	NC	NC
*D*-Glucopyranoside	12.61	1733	2.41 ± 0.01	ND	ND	NC	NC
Xylitol ^f^	12.70	1740	4.43 ± 0.67	ND	0.26 ± 0.04	0.0004	0.0006
Fucose	12.75	1742	Trace	ND	ND	NC	NC
Putrescine ^e^	12.90	1752	2.98 ± 0.21	146.29 ± 6.89	2.57 ± 2.78	< 0.0001	0.0003
*α*-Glycerophosphate ^f^	13.20	1771	4.38 ± 0.31	ND	0.77 ± 0.30	0.0001	0.0002
*α*-D-Mannopyranoside ^f^	13.30	1780	ND	5.58 ± 0.16	0.06 ± 0.03	< 0.0001	0.0003
Ethanolamine phosphate ^f^	13.40	1786	ND	4.09 ± 0.30	0.02 ± 0.01	< 0.0001	0.0002
Azelaic acid	13.58	1797	0.08 ± 0.00	ND	ND	NC	NC
Shikimic acid	13.69	1813	0.11 ± 0.01	ND	ND	NC	NC
Citric acid ^e^	13.80	1830	0.05 ± 0.01	8.30 ± 0.22	0.09 ± 0.03	< 0.0001	0.0002
*β*-_L_-Arabinofuranose	13.89	1836	3.30 ± 0.03	ND	ND	NC	NC
_L_-Arginine	13.90	1819	Trace	ND	ND	NC	NC
Unknown sugar *m/z* 319	14.01	1852	6.86 ± 0.33	ND	ND	NC	NC
Unknown *m/z* 173/316	14.05	1855	14.38 ± 0.59	ND	ND	NC	NC
*iso*-Citric acid	14.08	1860	Trace	ND	ND	NC	NC
Tetradecanoic acid	14.11	1865	Trace	ND	ND	NC	NC
Quinic acid	14.21	1878	0.03 ± 0.00	ND	ND	NC	NC
Fructose ^e^	14.34	1906	29.50 ± 15.00	1.51 ± 0.03	11.58 ± 5.83	0.0271	0.0305
Mannose ^f^	14.56	1918	Trace	ND	0.79 ± 0.54	0.0010	0.0012
Galactose	14.58	1925	4.51 ± 0.03	ND	ND	NC	NC
Glucose ^e^	14.60	1929	754.90 ± 368.30	1.45 ± 0.04	17.41 ± 10.67	0.0076	0.0094
Glucitol ^e^	14.88	1939	40.78 ± 2.90	0.36 ± 0.00	0.26 ± 0.04	< 0.0001	0.0002
Mannitol ^f^	14.95	1945	Trace	ND	0.96 ± 0.53	0.0347	0.0368
Tyrosine	15.10	1958	0.95 ± 0.02	ND	ND	NC	NC
Unknown sugar alcohol	15.13	1960	3.41 ± 0.02	ND	ND	NC	NC
*Chiro*-Inositol ^f^	15.15	1962	7.16 ± 0.68	ND	1.78 ± 0.69	0.0007	0.0009
Unknown sugar	15.17	1970	ND	ND	0.10 ± 0.14	NC	NC
Allonic acid GL	15.17	1970	0.81 ± 0.04	ND	ND	NC	NC
*N*-Acetyl-Glucosamine	15.30	1974	Trace	ND	ND	NC	NC
α-D-Glucopyranose	15.32	1976	ND	ND	0.05 ± 0.03	NC	NC
Gluconic acid	15.52	1992	ND	ND	1.59 ± 0.40	NC	NC
Unknown *m/z* 145/284	15.69	2006	7.19 ± 0.31	ND	ND	NC	NC
*Scyllo*-Inositol ^f^	15.83	2025	29.97 ± 1.35	ND	0.97 ± 0.28	< 0.0001	0.0002
*α*-D-Galactoside ^f^	15.86	2031	3.81 ± 0.09	ND	0.06 ± 0.02	< 0.0001	0.0002
Glucaric acid	16.35	2069	ND	0.15 ± 0.01	ND	NC	NC
*Myo*-Inositol ^f^	16.45	2086	274.54 ± 10.34	ND	1.36 ± 0.24	< 0.0001	0.0002
Oleic acid	17.90	2209	6.22 ± 0.37	ND	ND	NC	NC
Sorbitol-6-phosphate	18.65	2274	ND	ND	0.09 ± 0.11	NC	NC
Inositol-2-phosphate	19.85	2386	Trace	ND	ND	NC	NC
Unknown disaccharide	21.02	2484	ND	0.16 ± 0.00	ND	NC	NC
Unknown *m/z* 456/513	21.24	2502	ND	0.52 ± 0.02	ND	NC	NC
Unknown disaccharide	21.76	2545	ND	0.32 ± 0.01	ND	NC	NC
Sucrose ^e^	21.89	2597	3.32 ± 0.18	0.59 ± 0.01	0.11 ± 0.01	< 0.0001	0.0002
Trehalose ^e^	22.78	2651	10.11 ± 0.93	17.87 ± 0.69	0.77 ± 0.42	< 0.0001	0.0002

^a^ Concentrations of different metabolites (mM) were calculated as the mean ± SD of three biological replicates (two technical replicates for each). Raw numerical data underlying the table are provided in Supplementary Table 4.

^b^ RT, Retention time (min).

^c^ Experimental linear retention indices (LRI) were calculated based on n-alkanes (C8-C26) injected on ZB-5MS (30 m × 0.25 mm × 0.25 µm) under the same conditions as derivatized samples.

^d^ *p*-values were adjusted for multiple comparisons using the Benjamini-Hochberg False Discovery Rate (FDR) correction, applied only to valid statistical tests. Comparisons that could not be performed due to missing treatment groups were excluded from the FDR adjustment.

^e^ Different letters indicate statistically significant differences among treatments, while the same letter signifies no significant differences between them based on one-way analysis of variance (ANOVA), followed by post-hoc pairwise comparisons using Tukey’s HSD (*p* ≤ 0.05).

^f^ *p* < 0.05 indicates statistically significant differences among treatments, while *p* > 0.05 indicates no significant differences using Student’s *t*-test.

^g^ ND: Not detected.

^h^ NC: Not calculated.

Key metabolites unique to each species included _L_-aspartic acid, glucaric acid, and three unknown metabolites in *G. mellonella*, 1-oxo-propanoic acid, erythritol, xylose, α-D-glucopyranose, gluconic acid, sorbitol-6-phosphate, and unknown sugar in *A. mellifera* (Fig. 2d). On the other hand, TMS-based derivatization showed that trehalose was consistently detected in all three insect species, however, the haemolymph of *G. mellonella* (17.87 ± 0.69 mM) contained significantly higher levels of trehalose compared with *D. citri* (10.11 ± 0.93 mM) and *A. mellifera* (0.77 ± 0.42 mM).

**Fig. 3:**
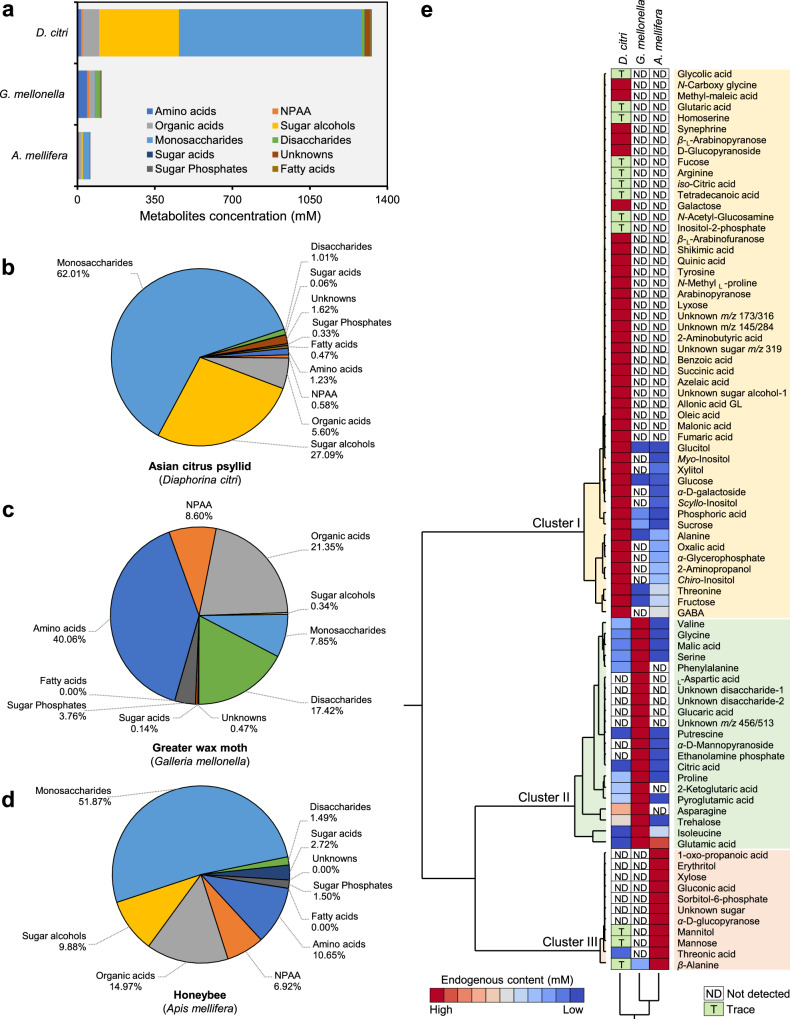
Haemolymph chemical composition of Asian citrus psyllid (*Diaphorina citri*), greater wax moth (*Galleria mellonella*), and honeybee (*Apis mellifera*) after TMS derivatization using GC-MS. **a** Concentration (mM) and chemical composition of different metabolic groups/classes detected in haemolymph of the three insect species (*n* = 3). **b**−**d** Percentage of different metabolic groups/classes detected in haemolymph of *D. citri*, *G. mellonella*, and *A. mellifera*, respectively. **e** Two-way hierarchical cluster analysis (HCA) of individual metabolites detected in the haemolymph of 3 insect species using GC-MS. The variation in the metabolite abundances between the three insect species is visualized in the heat-map diagram. Rows represent individual metabolites, while columns represent the insect species. Metabolites and treatments were organized using HCA based on similarities in auto-scaled values and correlations, respectively, with 95% confidence between groups from the discriminant function analysis, to construct the similarity dendrograms. Raw numerical data underlying the graph are provided in Supplementary Data 4. ND: Not detected, T: trace.

Moreover*, D. citri* exhibited a significantly higher total metabolite concentration compared to the other insects (Fig. 3a), with monosaccharides (62.01%) and sugar alcohols (27.09%), being the dominant components (Fig. 3b) but minimal content of organic acid (5.60%), amino acid (1.23%), and non-protein amino acids (NPAA; 0.58%). However, the haemolymph of *G. mellonella* and *A. mellifera* displays a more balanced distribution of amino acids, organic acids, and sugars (Fig. 3c, d, respectively). For instance, *G. mellonella* had a more diverse metabolic profile, with amino acids (40.06%) and organic acids (21.35%) being the most abundant metabolite classes (Fig. 3c). Additionally, it has a significant proportion of disaccharides (17.42%) and NPAA (8.60%), indicating broader metabolic flexibility. On the other hand, *A. mellifera* fell between these extremes with a high monosaccharide content (51.87%), moderate organic acids (14.97%), and amino acids (10.65%), along with a notable fraction of sugar alcohols (9.88%) and NPAA (6.92%) (Fig. 3d). Notably, the wax moth has the highest NPAA content (8.60%), followed by the honeybee (6.92%) and the psyllid (0.58%).

Additionally, the two-way hierarchical clustering analysis of detected metabolites across the three insect species highlighted differential metabolic signatures (Fig. 3e) with three distinct clusters. *D. citri* was clustered separately from the other two species within ‘Cluster I’ with 50 metabolites that were higher or existed only within its haemolymph. ‘Cluster II’ included 21 metabolites that were higher in the haemolymph of *G. mellonella*. For instance, TMS-based derivatization revealed the presence of trehalose in the haemolymph of all three insect species; however, *G. mellonella* exhibited markedly elevated levels compared to both *D. citri* and *A. mellifera*. Nevertheless, ‘Cluster III’ included only 11 metabolites that were unique for *A. mellifera*, but not other insect species, except for some traces in *D. citri* (Fig. 3e).

### *G. mellonella* haemolymph had higher amino acids and organic acids than *D. citri*

**Fig. 4:**
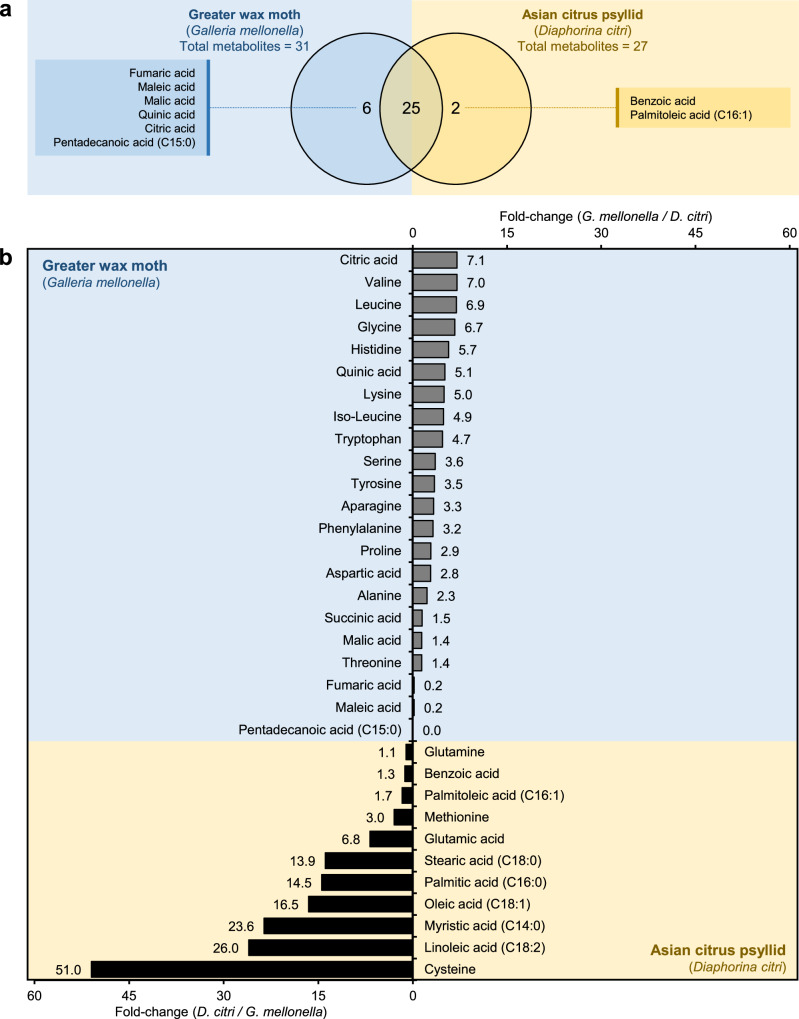
Haemolymph chemical composition of Asian citrus psyllid (*Diaphorina citri*) and greater wax moth (*Galleria mellonella*), after derivatization with methyl chloroformate (MCF) using GC-MS. **a** Venn Diagram of shared metabolites between *D. citri* and *G. mellonella* (*n* = 3). **b** Fold-change of individual metabolites detected in the haemolymph of *D. citri* and *G. mellonella* using GC-MS after MCF derivatization. Raw numerical data underlying the bar graphs are provided in Supplementary Data 5 and 6.

Generally, MCF-based comparative haemolymph metabolic profiling revealed the superiority of *G. mellonella* over *D. citri* in most amino acids and organic acids content (Table 2 and Fig. 4). Although 33 metabolites were detected after MCF derivatization, only 25 core metabolites were common in both *D. citri* and *G. mellonella* hemolymphs (Table 2 and Fig. 4a). In contrast to the TMS derivatization results, *G. mellonella* haemolymph contained a greater variety of carboxylic compounds, with 31 metabolites detected compared to 27 in *D. citri*. It is worth mentioning that six metabolites, mainly associated with the TCA cycle, including fumaric acid, maleic acid, malic acid, quinic acid, citric acid, and pentadecanoic acid, were detected exclusively in *G. mellonella*, while only two, benzoic acid and palmitoleic acid, were unique to *D. citri* (Fig. 4a).

**Table 2:** Hemolymph chemical composition of Asian citrus psyllid (*Diaphorina citri*) and greater wax moth (*Galleria mellonella*) after MCF derivatization using GC-MS (*n* = 3) ^a^

MCF metabolite	RT ^b^	LRI ^c^	Asian citrus psyllid (*Diaphorina citri*)	Greater wax moth (*Galleria mellonella*)	*p*-value ^d^	FDR-adjusted *p*-value ^e^
Fumaric acid	6.71	603	ND ^f^	0.22 ± 0.02	NC ^g^	NC
Maleic acid	6.77	608	ND	0.22 ± 0.02	NC	NC
Succinic acid	6.94	614	0.46 ± 0.11	0.69 ± 0.08	0.0429	0.0536
Benzoic acid	8.50	1065	0.78 ± 0.23	ND	NC	NC
Glycine	8.88	1116	0.77 ± 0.19	5.16 ± 0.93	0.0013	0.0046
_L_-Alanine	8.90	1116	4.56 ± 1.23	10.36 ± 1.76	0.0095	0.0148
_L_-Valine	10.89	1286	1.02 ± 0.17	7.17 ± 1.40	0.0016	0.0044
_L_-Leucine	12.13	1382	0.77 ± 0.15	5.34 ± 1.21	0.0029	0.0073
_L_-Isoleucine	12.31	1397	0.51 ± 0.06	2.50 ± 0.57	0.0039	0.0089
_L_-Threonine	12.38	1406	0.29 ± 0.07	0.41 ± 0.12	0.2090	0.2177
Malic acid	12.58	1423	ND	1.43 ± 0.21	NC	NC
_L_-Proline	12.77	1433	11.26 ± 1.75	32.50 ± 4.42	0.0015	0.0047
_L_-Asparagine	12.74	1440	0.82 ± 0.28	2.71 ± 1.28	0.0669	0.0796
Quinic acid	13.30	1489	ND	5.12 ± 2.86	NC	NC
Aspartic acid	13.68	1514	0.12 ± 0.06	0.34 ± 0.24	0.1983	0.2155
Citric acid	13.72	1527	ND	7.05 ± 2.06	NC	NC
_L_-Serine	14.56	1584	1.56 ± 0.54	5.58 ± 1.38	0.0093	.0155
_L_-Glutamine	14.56	1627	8.14 ± 2.05	7.58 ± 2.68	0.7880	0.7880
Glutamic acid	15.22	1637	3.33 ± 0.34	0.49 ± 0.30	0.0004	0.0025
_L_-Methionine	15.40	1650	0.62 ± 0.20	0.21 ± 0.06	0.0273	0.0359
_L_-Cysteine	16.45	1733	0.51 ± 0.23	0.01 ± 0.01	0.0197	0.0274
_L_-Phenylalanine	16.80	1755	0.49 ± 0.16	1.58 ± 0.35	0.0080	0.0143
Myristic acid (C14:0)	16.87	1765	1.18 ± 0.13	0.05 ± 0.02	0.0001	0.0025
Pentadecanoic acid (C15:0)	17.94	1850	ND	0.01 ± 0.01	NC	NC
Palmitoleic acid (C16:1)	18.60	1907	0.59 ± 0.22	ND	NC	NC
Palmitic acid (C16:0)	18.98	1942	8.25 ± 0.82	0.57 ± 0.30	0.0001	0.0013
_L_-Lysine	19.80	2009	1.09 ± 0.63	5.43 ± 1.36	0.0074	0.0142
_L_-Histidine	20.44	2054	0.18 ± 0.19	1.03 ± 0.26	0.0102	0.0150
Linoleic acid (C18:2)	20.59	2072	8.33 ± 1.38	0.32 ± 0.15	0.0006	0.0030
Oleic acid (C18:1)	20.66	2078	3.64 ± 0.72	0.22 ± 0.06	0.0012	0.0050
Stearic acid (C18:0)	20.88	2097	6.95 ± 0.62	0.50 ± 0.38	0.0001	0.0008
_L_-Tyrosine	21.43	2133	0.97 ± 0.3	3.35 ± 0.69	0.0054	0.0113
_L_-Tryptophan	23.14	2266	0.15 ± 0.00	0.71 ± 0.45	0.0974	0.1107

^a^ Concentrations of different metabolites (mM) were calculated as the mean ± SD of three biological replicates (two technical replicates for each). Raw numerical data underlying the table are provided in Supplementary Table 5.

^b^ RT, Retention time (min).

^c^ Experimental linear retention indices (LRI) were calculated based on n-alkanes (C8-C26) injected on ZB-5MS (30 m × 0.25 mm × 0.25 µm) under the same conditions as derivatized samples.

^d^ *p* < 0.05 indicates statistically significant differences among treatments, while *p* > 0.05 indicates no significant differences using Student’s *t*-test.

^h^ *p*-values were adjusted for multiple comparisons using the Benjamini-Hochberg False Discovery Rate (FDR) correction, applied only to valid statistical tests. Comparisons that could not be performed due to missing treatment groups were excluded from the FDR adjustment.

^e^ ND: Not detected.

^**f**^ NC: not calculated.

Generally, *G. mellonella* haemolymph exhibited higher levels of amino acids and organic acids, such as citric acid (7.05 ± 2.06 mM), which was not detected in *D. citri*. Moreover, *G. mellonella* had significantly higher levels of the most abundant amino acid _L_-proline (32.50 ± 4.42 mM) than *D. citri* (11.26 ± 1.75 mM) (Table 2). However, both insects had almost the same levels of _L_-glutamine (8.14 ± 2.05 and 7.58 ± 2.68 mM) without significant differences between them. On the other hand, *D. citri* showed a pronounced enrichment of fatty acids and sulfur-containing compounds, such as linoleic acid, palmitic acid, and cysteine (Table 2). In other words, *G. mellonella* haemolymph had higher levels of the essential nutritional needs of ‘*Ca*. L. asiaticus’ mainly amino acids and organic acids, such as citric acid (7.1-fold), valine (7.0-fold), leucine (6.9-fold), glycine (6.7-fold), and histidine (5.7-fold), while *D. citri* had elevated levels of cysteine showing the highest fold-change (51.0-fold), followed by linoleic acid (26.0-fold), myristic acid (23.6-fold), palmitic acid (16.5-fold), oleic acid (14.5-fold), and stearic acid (13.9-fold) (Fig. 4b). It is worth mentioning that after applying the Benjamini-Hochberg-based FDR correction, only succinic acid was statistically insignificant (*q* = 0.0536) and did not meet the adjusted significance threshold (*q* < 0.05), indicating its low robustness in this dataset (Table 2).

### BF_3_-based derivatization reveals a pronounced free fatty acids profile from ***D. citri*** haemolymph

Generally, twelve free fatty acids were detected in both insect species after derivatization with BF_3_ (Table 3). While *G. mellonella* exhibited a slightly diverse free fatty acids profile, with 10 metabolites (three of which were detected exclusively within its haemolymph), *D. citri* possessed nine metabolites with only two unique compounds. Moreover, *D. citri* had significantly higher levels of all seven shared fatty acids (Table 3). Oleic acid (35.74 ± 22.13 mM) was the most abundant fatty acid detected in both insect species, followed by stearic acid (10.70 ± 6.25 mM) and palmitic acid (5.73 ± 3.25 mM). In *D. citri* haemolymph, palmitoleic acid, lauric acid, myristic acid, and arachidic acid were also detected, but in lower concentrations (<1 mM). Moreover, linoleic acid and linolenic acid were also detected, but in trace amounts (Table 3). It is worth mentioning that After applying the Benjamini-Hochberg-based FDR correction, only oleic acid (C18:1(Z)) remained statistically significant (*q* = 0.0499), while the other fatty acids did not meet the adjusted significance threshold (*q* < 0.05), indicating that oleic acid is the most robustly differentially abundant fatty acid in this dataset (Table 3).

**Table 3:** Concentration of different free fatty acids (mM) detected in the hemolymph of greater wax moth (*Galleria mellonella*) larvae and Asian citrus psyllid (*Diaphorina citri*) after derivatization with boron trifluoride-methanol using GC-MS (*n* = 3)

Fatty acid	RT ^a^	Asian citrus psyllid (*Diaphorina citri*) ^b^	Greater wax moth (*Galleria mellonella*) ^b^	*p*-value ^c^	FDR-adjusted *p*-value ^d^
Lauric acid (C12:0)	14.45	0.17 ± 0.14	ND ^**e**^	NC ^**f**^	NC
Myristic acid (C14:0)	15.70	0.04 ± 0.02	0.003 ± 0.001	0.0329	0.0823
Pentadecanoic acid (C15:0)	16.75	ND	0.002 ± 0.000	NC	NC
Palmitoleic acid (C16:1)	17.50	0.70 ± 0.35	0.017 ± 0.004	0.0278	0.1390
Palmitic acid (C16:0)	17.74	5.73 ± 3.25	0.274 ± 0.070	0.0438	0.0548
Linolenic acid (C18:3)	19.37	Trace	0.005 ± 0.001	NC	NC
Linoleic acid (C18:2)	19.37	Trace	0.095 ± 0.020	NC	NC
Oleic acid (C18:1(Z))	19.45	35.74 ± 22.13	0.249 ± 0.030	0.0499	0.0499
Eliadic acid (C18:1 (E))	19.50	ND	0.016 ± 0.003	NC	NC
Stearic acid (C18:0)	19.68	10.70 ± 6.25	0.046 ± 0.004	0.0418	0.0697
Arachidic acid (C20:0)	23.03	0.39 ± 0.25	ND	NC	NC
Hexanedioic acid, dioctyl ester (Adiimoll; C22)	21.89	ND	0.013 ± 0.001	NC	NC

^a^ RT, Retention time (min).

^b^ Concentrations of different fatty acids (mM) were calculated as the mean ± SD of three biological replicates (two technical replicates for each).

^c^ *p* < 0.05 indicates statistically significant differences among treatments, while *p* > 0.05 indicates no significant differences using Student’s *t*-test.

^d^ *p*-values were adjusted for multiple comparisons using the Benjamini-Hochberg False Discovery Rate (FDR) correction, applied only to valid statistical tests. Comparisons that could not be performed due to missing treatment groups were excluded from the FDR adjustment.

^**e**^ ND: not detected.

^**f**^ NC: not calculated.

### Haemolymph of ***G. mellonella*possesses higher** nucleotides and sugar-nucleotide composition than ***D. citri***

**Fig. 5:**
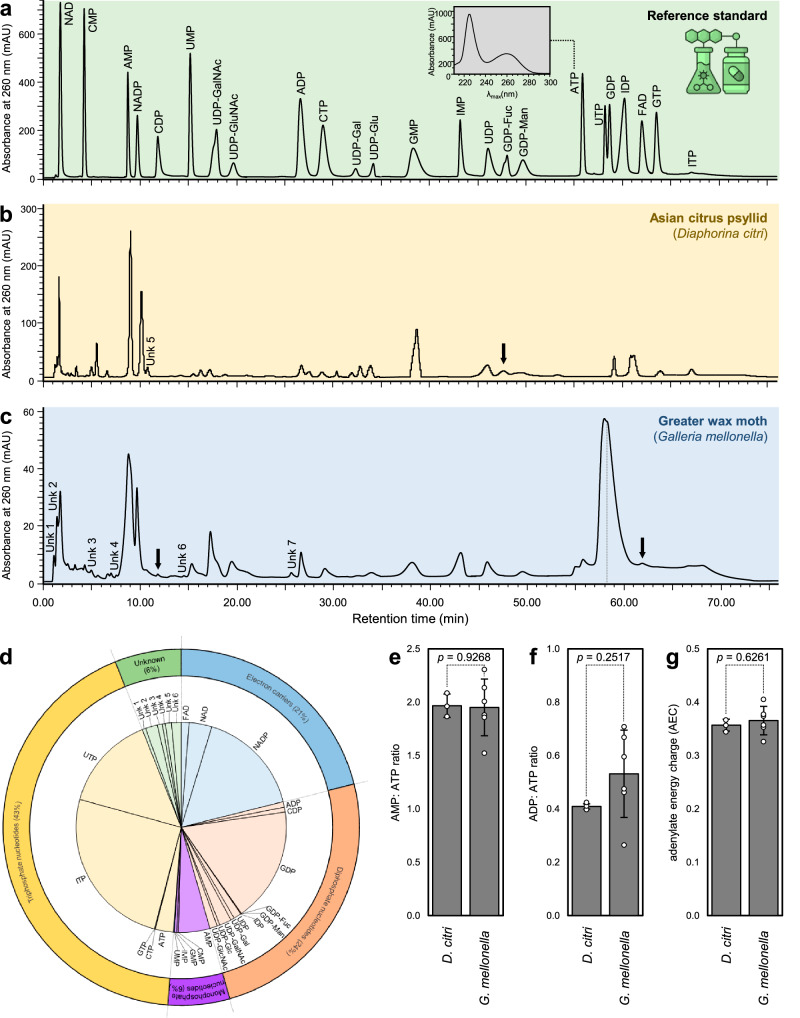
Representative HPLC chromatograms of nucleotides and sugar-nucleotides detected in the haemolymph of Asian citrus psyllid (*Diaphorina citri*), and greater wax moth (*Galleria mellonella*). **a** Reference standard, (**b**) Haemolymph of Asian citrus psyllid (*Diaphorina citri*), (**c**) Haemolymph of Greater wax moth (*Galleria mellonella*), (**d**) Percentage of different nucleotides and sugar-nucleotides and their energetic groups detected in haemolymph of *G. mellonella*. Raw numerical data underlying the pie chart are provided in Supplementary Data 7. **e**−**g** AMP: ATP ratio, ADP: ATP ratio, and adenylate energy charge (AEC), respectively, of *D. citri* (*n* = 3) and *G. mellonella* (*n* = 6). Bars and error bars represent means and SDs, respectively. *p* < 0.05 indicates statistically significant differences among treatments, whereas *p* > 0.05 signifies no significant differences between them using the Student *t*-test. Raw numerical data underlying the bar graph are provided in Supplementary Data 8.

Comparable HPLC chromatograms of nucleotides and sugar-nucleotide profiles between the reference standards (Fig. 5a), *D. citri* (Fig. 5b), and *G. mellonella* (Fig. 5c) haemolymph were analyzed and quantified. Generally, 30 nucleotides and sugar-nucleotides were detected using HPLC (Table 4); however, only 21 core nucleotides and sugar-nucleotides were found to be common between *G. mellonella* and *D. citri* (Table 4). In *G. mellonella* haemolymph, triphosphate nucleotides were the most dominant class (43%), followed by diphosphate nucleotides and electron carriers (24 and 21%, respectively); however, monophosphate nucleotides and unknown nucleotides were minimal with almost similar proportions (6% each class) (Fig. 5d).

**Table 4:** Concentration (ng.*μ*L^-1^) of different nucleotides and sugar-nucleotides detected in the hemolymph of greater wax moth (*Galleria mellonella*) larvae and Asian citrus psyllid (*Diaphorina citri*) using HPLC (*n* = 3)

Compound ^a^	Abbreviation	Asian citrus psyllid (*Diaphorina citri*) ^b,c^	Greater wax moth (*Galleria mellonella*) ^b^
Unknown 1	UK1	2.5 ± 0.5	8.7 ± 2.6
Unknown 2	UK2	ND ^d^	30.0 ± 7.2
*β*-Nicotinamide adenine dinucleotide hydrate	NAD	14.0 ± 2.0	56.8 ± 15.2
cytidine 5′-monophosphate	CMP	Trace	3.7 ± 2.8
Unknown 3	UK3	4.5 ± 1.0	11.9 ± 4.2
Unknown 4	UK4	ND	7.8 ± 2.9
Adenosine 5′-monophosphate	AMP	13.9 ± 1.0	78.3 ± 16.8
*β*-Nicotinamide adenine dinucleotide phosphate, oxidized form	NADP	4.0 ± 1.9	265.6 ± 108.7
Unknown 5	UK5	19.9 ± 1.8	ND
Cytidine-5′-diphosphate	CDP	ND	11.9 ± 1.8
Unknown 6	UK6	ND	15.1 ± 7.2
Uridine 5′-monophosphate	UMP	Trace ^e^	2.3 ± 0.9
Uridine 5′-diphospho-*N*-acetyl-d-galactosamine	UDP-GalNAc	Trace	29.8 ± 3.6
Uridine 5′-diphospho-*N*-acetyl-d-glucosamine	UDP-GlcNAc	6.6 ± 0.5	18.9 ± 3.2
Unknown 7	UK7	ND	24.1 ± 12.2
Adenosine 5′-diphosphate	ADP	2.9 ± 0.3	13.8 ± 8.2
Cytidine-5′-triphosphate	CTP	3.8 ± 0.4	2.3 ± 1.0
Uridine 5′-diphospho-d-galactose	UDP-Gal	2.4 ± 0.7	9.2 ± 3.3
Uridine 5′-diphospho-d-glucose	UDP-Glc	5.2 ± 0.9	6.9 ± 1.7
Guanosine 5′-monophosphate	GMP	2.9 ± 0.2	Trace
Inosine-5′-monophosphate	IMP	3.0 ± 0.5	6.1 ± 1.1
Uridine 5′-diphosphate	UDP	15.7 ± 2.6	19.3 ± 5.4
Guanosine 5′-diphosphate-beta-l-fucose	GDP-Fuc	12.5 ± 2.1	ND ^d^
Guanosine 5′-diphosphate-d-mannose	GDP-Man	2.2 ± 0.5	1.8 ± 0.6
Adenosine 5′-triphosphate	ATP	7.1 ± 0.9	45.0 ± 13.3
Uridine 5′-triphosphate	UTP	5.9 ± 1.1	238.3 ± 22.2
Guanosine 5′-diphosphate	GDP	4.5 ± 1.4	285.8 ± 27.0
Flavin adenine dinucleotide	FAD	ND	19.6 ± 15.6
Guanosine 5′-triphosphate	GTP	14.3 ± 1.2	ND
Inosine-5′-triphosphate	ITP	2.4 ± 1.0	408.1 ± 82.3

^a^ The unknown compounds were tentatively quantified using the peak area of the ATP authentic standard.

^**b**^ Concentrations of different nucleotides and sugar-nucleotides were calculated as the mean ± SD of three biological replicates (two technical replicates for each).

^**c**^ Based on previously published data (Killiny et al.^62^).

^**d**^ ND: not detected.

^**e**^ Trace means <1 ng.*μ*L^-1^.

Triphosphate nucleotides of *G. mellonella* included the most abundant nucleotides ITP (25%), UTP (15%), ATP (3%), and some trace of CTP and GTP. On the other hand, diphosphate nucleotides included five nucleotides (ADP, CDP, GDP, IDP, and UDP) and six sugar-nucleotides (GDP-Fuc, GDP-Man, UDP-Gal, UDP-GalNAc, UDP-Glc, and UDP-GlcNAc) with superiority of GDP within this class (Fig. 5d). Electron carriers of *G. mellonella* included NADP, NAD, and FAD. Moreover, several unknown compounds (UK1–UK7) were detected, with some present in one species but absent in the other. Notably, *D. citri* lacked detectable levels of CDP, FAD, and four unknown compounds, while *G. mellonella* exhibited high concentrations of these metabolites.

Although *G. mellonella* haemolymph had higher levels of AMP (78.3 ± 16.8 ng.*μ*L^-1^), ADP, and ATP than *D. citri* (Table 4), the AMP-to-ATP (Fig. 5e) and ADP-to-ATP (Fig. 5f) ratios were almost the same with no significant differences between *D. citri* and *G. mellonella* (*p* = 0.9268 and 0.2517, respectively), indicating a stable adenylate energy charge (AEC) between both insect species (Fig. 5g).

**Fig. 6:**
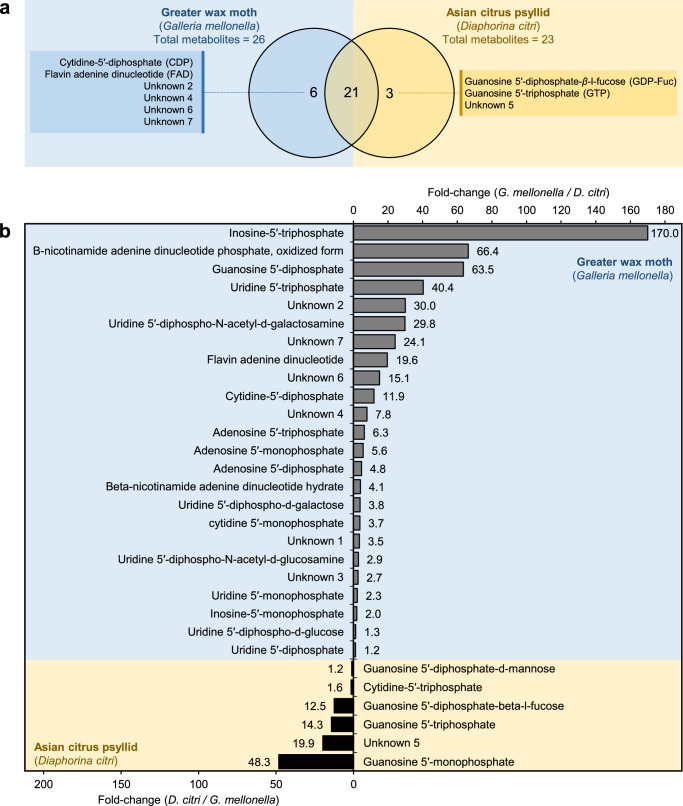
Nucleotides and sugar-nucleotides detected in the haemolymph of Asian citrus psyllid (*Diaphorina citri*) and greater wax moth (*Galleria mellonella*), using HPLC. **a** Venn Diagram of shared nucleotides and sugar-nucleotides between *D. citri* and *G. mellonella* (*n* = 3). **b** Fold-change of individual nucleotides and sugar-nucleotides detected in the haemolymph of *D. citri* and *G. mellonella* using HPLC. Raw numerical data underlying the bar graph are provided in Supplementary Data 9.

The haemolymph of *G. mellonella* demonstrated greater nucleotide and sugar-nucleotide diversity, comprising 27 distinct compounds, including six that were uniquely detected in this species. (Table 1 and Fig. 6a) including CDP, FAD, and four unknown nucleotides/sugar-nucleotide, whereas the haemolymph of *D. citri* possessed 24 nucleotides and sugar-nucleotide, with only three of them were detected exclusively from its haemolymph, including GTP, GDP-Fuc, and one unknown metabolite (Table 1 and Fig. 6a). Additionally, the fold-change analysis showed that *G. mellonella* had significantly higher levels of nucleotides, particularly ITP (170-fold), oxidized NADP (66.4-fold), and GDP (63.5-fold). However, *D. citri* exhibited higher levels of GMP (48.3-fold), GDP-Man (1.6-fold), and GDP-Fuc (12.5-fold) (Fig. 6b).

## Discussion

Culturing *Liberibacter* species, in particular ‘*Ca*. L. asiaticus’, remains a key challenge in disease management, as well as pathogen nomenclature, despite significant research efforts. Due to the requirements of specific conditions, ‘*Ca*. L. asiaticus’ has never been studied in axenic culture^18^ and is still under the ‘*Candidatus’* taxa. While transient ‘*Ca*. L. asiaticus’ growth has been reported on culture media containing citrus vein extract^10^, citrus Juice^11^, insect cell cultures^33^, or feeder cells^34^; none of them has achieved sustained propagation of ‘*Ca*. L. asiaticus’ in axenic culture yet. Accordingly, the sole culturable relative, *L. crescens*, has emerged as the only model for studying ‘*Ca*. Liberibacter’ species and to understand its biology^35–39^. Although the essential genes required for *L. crescens* culture were identified previously, particularly those involved in amino acid biosynthesis, stress response, and transporters^36^, genes are missing in ‘*Ca*. L. asiaticus’. This might be mainly due to the highly reduced genome of ‘*Ca*. L. asiaticus’^8,40,41^, which suggests a strong dependence on host-derived nutrients. Moreover, *L. crescens* was introduced as a cultured surrogate for the functional genomics of uncultured ‘*Ca. Liberibacter*’ species^37^. However, *L. crescens* does not fully replicate the distinctive characteristics and nutritional needs of *‘Ca*. L. asiaticus’, which highlights the urgent need for alternative models (s) that can support the growth and viability of ‘*Ca*. L. asiaticus’ and facilitate its study under laboratory conditions.

Previous studies showed that the hemipteran striped mealybug can acquire and retain *‘Ca*. L. asiaticus’ after feeding on infected plant tissue^17^. These findings closely align with our study’s central premise that *G. mellonella*, an alternative lepidopteran model, can transiently support short-term persistence of *‘Ca*. L. asiaticus’. Although the outcome may differ in terms of pathogenicity or replication efficiency, both studies reinforce the idea that host biology, metabolome, and microbiome context shape *‘Ca*. L. asiaticus’ viability^17^, and support the utility of alternative models for understanding *‘Ca*. L. asiaticus’ dynamics and facilitating antimicrobial screening. Notably, while *‘Ca*. L. asiaticus’ was able to translocate within the mealybug from the gut to the salivary glands; it failed to induce disease symptoms when transmitted to citrus plants^17^, suggesting vector-specific genotypic selection may influence pathogen virulence and host-pathogen compatibility. We believe that vector-specific selection may influence pathogen behavior and virulence; therefore, further genotypic analysis studies are required to evaluate whether ‘*Ca*. L. asiaticus’ populations in *G. mellonella* undergo a similar selection that could affect their suitability for downstream applications.

The replication kinetics of *‘Ca*. L. asiaticus’ remains a significant challenge in the study of its pathobiology, particularly due to the pathogen’s unculturable nature and its dependence on host environments. For example, *L. crescens*, a culturable close relative of *‘Ca*. L. asiaticus’, was reported to have a doubling time of 36.7 h of incubation in liquid BM7 medium at 28°C^20^. However, recent studies suggest that *‘Ca*. L. asiaticus’ replicates even more slowly within its insect host^42^, and *in planta*^43^. For instance, *‘Ca*. L. asiaticus’ replication within its psyllid vector *D. citri* was proven to proceed at a low rate, with the pathogen remaining at relatively stable titers for extended periods^42^. Similarly, it was reported previously that *‘Ca*. L. asiaticus’ titers initially declined after psyllid inoculation in new shoots of *C. sinensis* (susceptible plant host), *Murraya paniculata* (partially resistant), and *Bergera koenigii* (fully resistant)^43^. However, it subsequently increased and established in *Citrus × sinensis* and *Murraya paniculata*, with sustained colonization only in the susceptible *C. × sinensis*^43^. Our findings in *Galleria mellonella* align with these earlier observations, reinforcing the concept that *‘Ca*. L. asiaticus’ does not rapidly replicate in vivo, even in permissive surrogate hosts. In our time-course experiment spanning 0 to 96 h post-inoculation, qPCR data revealed stable bacterial titers through 48–72 h, followed by a decline at 96 h. While the persistence of detectable *‘Ca*. L. asiaticus’ DNA suggests viability within the larval hemocoel during this window; the slow doubling rate observed in previous studies implies that only minimal, if any, replication may have occurred within the time frame of our experiment.

Larvae of *G. mellonella* were previously proposed as an alternative model host for several human pathogens^24,25,44^, and insect-pathogenic fungi^45^. However, to the best of my knowledge, it has never been reported to host plant pathogens, except the trans-kingdom pathogen *Fusarium oxysporum* isolate FGSC 9935^23^, which was previously reported to cause vascular wilt on tomato plants and as an immunosuppressor of mice^46^. In the current study, the temporal dynamics of *‘Ca*. L. asiaticus’ persistence within *G. mellonella* larvae, as revealed by qPCR analysis, offers important insights into the potential of this lepidopteran model for short-term pathogen studies. Following inoculation with *‘Ca*. L. asiaticus’-positive *C. sinensis* phloem sap or *D. citri* haemolymph, larvae maintained high bacterial titers through 48−72 h hpi, suggesting that the bacterium can persist within the larval hemocoel for at least two to three days. This early persistence phase likely reflects a combination of initial bacterial load and host permissiveness, possibly influenced by the nutrient-rich composition of the *G. mellonella* hemolymph, which includes elevated levels of ATP and essential amino acids necessary for *‘Ca*. L. asiaticus’ survival^4,29^.

The subsequent increase in *Ct* values observed between 72 and 96 hpi, indicative of a decline in bacterial titer, may result from the activation of the host’s innate immune defenses, particularly melanization and antimicrobial peptide expression, which are well-characterized responses in *G. mellonella*^28^. These results highlight the potential of *G. mellonella* as a short-term in vivo model for studying *‘Ca*. L. asiaticus’ infection dynamics and evaluating antimicrobial strategies. However, the decline in bacterial persistence beyond 72 h suggests that while *G. mellonella* can transiently host *‘Ca*. L. asiaticus’, it may not support long-term proliferation without further optimization. Future work aimed at modulating immune responses or refining the inoculation conditions could enhance the utility of this model for extended studies. Moreover, given that ‘*Ca*. L. asiaticus’-infected phloem sap exhibits a markedly altered metabolic profile compared to non-infected (healthy) sap^47–49^, observed differences in host survival may be attributed, at least in part, to indirect effects such as the accumulation of reactive oxygen species (ROS), which are known to increase in infected citrus tissues. Future studies should include filtration-based removal of ‘*Ca*. L. asiaticus’ from infected sap to differentiate between bacterial pathogenicity and host-derived toxic compounds, such as ROS.

The biological differences between *G. mellonella* and the natural psyllid vector of *‘Ca*. L. asiaticus’ (*D. citri*) may significantly influence infection outcomes and must be carefully considered when interpreting the utilization of this surrogate model. Unlike *D. citri*, *G. mellonella* possesses a hemocoel-based circulatory system and a relatively simplified innate immune system that includes cellular responses (e.g., phagocytosis, encapsulation, and nodulation) and humoral components such as antimicrobial peptides, phenoloxidase activity, and melanization^28^. The rapid melanization observed upon *‘Ca*. L. asiaticus’ challenge in *G. mellonella* indicates activation of the prophenoloxidase cascade, a defense mechanism that does not exist in the *D. citri* hemolymph. This innate immune response may limit long-term pathogen persistence and explains the observed decline in bacterial titers after 48−72 hpi. Accordingly, the rapid mortality of *G. mellonella* upon ‘*Ca*. L. asiaticus’ infection limits the utility of the model for long-term experiments beyond 4 days. However, its value for short-term infection assays and antimicrobial screening remains substantial. Optimization of inoculum concentration and environmental conditions may extend the survival of *G. mellonella*. Moreover, further studies are required to explore strategies such as immune modulation and dietary supplementation to extend the viability of *G. mellonella* as a host beyond the current four-day window.

As an intracellular, fastidious bacterial pathogen with a highly reduced genome that lacks several essential metabolic-associated genes^8,40,41^, ‘*Ca*. L. asiaticus’ depends on its host for essential nutrients and energy^29^. For example, genome-wide analysis showed that ‘*Ca*. L. asiaticus’ lacks a functional glycolysis pathway and the glyoxalase system, and cannot independently produce ATP^29^. As an alternative, it depends on an ATP translocase to sneak energy directly from its host cells^50^ making it an energy scavenger^29^. Accordingly, a good alternative model host should supply ATP or a highly bioavailable energy source that could be absorbed and utilized directly by ‘*Ca*. L. asiaticus’. Previously, we showed that the haemolymph of *D. citri* contains more than 20 nucleotides and sugar nucleotides^32^. In agreement with these findings, our HPLC-based studies showed that the haemolymph of *G. mellonella* has higher nucleotides and sugar-nucleotides composition than *D. citri*, with about 27 detected compounds. Collectively, these findings suggest that while ‘*Ca*. L. asiaticus’ has a functional ATP translocase^50^, *G. mellonella* possesses the essential nucleotides to host with <6-fold higher ATP that might support its energetic needs.

During the past decade, several trials have been done to develop a chemically defined medium that satisfies the nutritional needs of ‘*Ca*. L. asiaticus’ to survive outside its natural environment^11–13^. Previously, we showed that citric acid was significantly increased in ‘*Ca*. L. asiaticus’-infected citrus leaves^4^, which was recently confirmed from infected fruits^51^. Moreover, the addition of commercial grapefruit juice to the King’s B media prolonged the viability of ‘*Ca*. L. asiaticus’ which might be due to its high citric acid content^11^. Accordingly, alternative model hosts should provide sufficient citrate as the primary carbon source for ‘*Ca*. L. asiaticus’. Interestingly, our MCF-based metabolic profiling revealed that citric acid was detected exclusively in *G. mellonella*, but not *D. citri*. These findings suggest that some key metabolites, such as citric acid and amino acids, are present in the haemolymph of *G. mellonella* and play a key role in ‘*Ca*. L. asiaticus’ survival^11^.

The genome of ‘*Ca*. L. asiaticus’ leaks for key biosynthetic pathways for amino acids, vitamins, and cofactors, as well as carbohydrate metabolism^8,40,41^. For instance, the biosynthesis pathways of tyrosine, phenylalanine, tryptophan, proline, cysteine, and histidine were absent from ‘*Ca*. L. asiaticus’ genome but were produced by *L. crescens* BT-1^20^. The absence of these pathways should not affect culturing as long as the growth medium or alternative model host contains all required amino acids^20^. Interestingly, our metabolic analysis showed that the haemolymph of *G. mellonella* exhibited higher levels of these amino acids. These findings suggest that *G. mellonella* might provide the essential amino acids that are required for ‘*Ca*. L. asiaticus’ persistence.

The idea that ‘*Ca*. L. asiaticus’ relies on host cells for essential metabolites^9^ and has been extended to include possible symbiotic interaction that provides essential nutrients since host-free cultures have been partially successful^14,15^. For example, the co-cultivation of ‘*Ca*. L. asiaticus’ with actinobacteria related to *Propionibacterium acnes*, enhanced its viability^9^. Furthermore, the microbial community surrounding ‘*Ca*. L. asiaticus’ directly affects its growth^15^, and modifying its associated microbiota negatively affects its survival^52^. Additionally, the endogenous microbiota of *G. mellonella* represents a critical, yet underexplored, factor that may shape infection dynamics. The larval gut and hemocoel harbor a diverse bacterial and fungal community, including members of *Enterococcus*, *Lactococcus*, and *Candida*, among others^53,54^. These microbial communities are known to interact with invading pathogens, either by direct antagonism or by modulating host immunity. For instance, certain gut-associated bacteria have been shown to induce systemic immune priming in *G. mellonella*, which could affect the survival and proliferation of *‘Ca*. L. asiaticus’ in this model^55^. Moreover, unlike *D. citri*, which harbors symbionts that may support *‘Ca*. L. asiaticus’ acquisition and colonization, *G. mellonella* lacks these co-evolved symbionts, possibly leading to different metabolic or immunological outcomes. Therefore, while *G. mellonella* serves as a valuable short-term infection model, the immunological and microbiological landscape it presents is distinct from that of *‘Ca*. L. asiaticus’s natural hosts, and this should be acknowledged in both interpretation and experimental design.

These findings suggest microbial interactions as another key factor in ‘*Ca*. L. asiaticus’ survival. In other words, a successful model host should have a specific microbiota that provides missing nutrients or bioactive molecules necessary for growth. Interestingly, *G. mellonella* possesses a highly diverse bacterial and fungal microbiome^53,54,56^ that is severely affected by diet, ontogeny^55^, or bacterial infection^53^. We suggest that co-cultivation of ‘*Ca*. L. asiaticus’ within the *G. mellonella* microbiome might help its persistence and viability. However, further studies are required to analyze the main species of *G. mellonella* microbiome and how ‘*Ca*. L. asiaticus’ could benefit from this microbial community.

## Conclusion

Building on these findings, the integration of *G. mellonella* as a surrogate host for *‘Ca*. L. asiaticus’ represents a significant advancement in host-pathogen-vector research, particularly in the context of unculturable and fastidious plant pathogens. The temporal persistence of *‘Ca*. L. asiaticus’ within *G. mellonella*, combined with its metabolically rich hemolymph, mirrors the bacterium’s intracellular nutritional requirements and highlights the model’s biological relevance. *G. mellonella* haemolymph provides favorable conditions for the viability, survival, and potential proliferation of ‘*Ca*. L. asiaticus’, include but are not limited to (i) high levels of ATP and energetic nucleotides as an alternative energy source, (ii) sufficient citrate levels as the primary carbon source, (iii) essential amino acids as the primary nitrogen source, and (vi) co-culture with a diverse microbiome that could provide other essential biological factors, to be determined yet, needed to sustain ‘*Ca*. L. asiaticus’ persistence.

Insights gained from the metabolomic profiling of *G. mellonella* haemolymph highlight its enriched composition in citrate, amino acids, and ATP. These metabolites are essential for *‘Ca*. L. asiaticus’ viability and energy acquisition. This biochemical richness likely contributes to the observed persistence of the pathogen within the larval host. Leveraging this information, the rational design of a synthetic or semi-defined culture medium that replicates key nutritional features of *G. mellonella* haemolymph may provide a promising foundation for developing an axenic system for *‘Ca*. L. asiaticus’ cultivation. Such a medium could serve as a crucial step toward overcoming one of the major barriers in *‘Ca*. L. asiaticus’ research, its in vitro unculturability. Moreover, given its scalability, easy handling, and low maintenance cost, *G. mellonella* presents a practical and accessible model for high-throughput screening of antimicrobial compounds targeting *‘Ca*. L. asiaticus’. Its larger size compared to traditional insect vectors, such as *D. citri*, allows for more precise experimental manipulation, including direct inoculation and sampling of hemolymph, making it particularly well-suited for laboratory-based assays. These advantages, coupled with its established use in microbial pathogenesis studies, position *G. mellonella* as a valuable surrogate host for short-term *‘Ca*. L. asiaticus’ infection models.

## Materials and methods

### Plant materials and growth conditions

The most common HLB-susceptible cultivar, ‘Valencia’ sweet orange (*Citrus sinensis*), was used as an experimental plant throughout this study. All trees were maintained under greenhouse conditions at 28 ± 1 °C, with 75 ± 5% RH, and an 8D:16 L photoperiod at the Citrus Research and Education Center (CREC-IFAS), University of Florida, Lake Alfred, Florida. To obtain the ‘*Ca*. L. asiaticus’-infected plant materials, 12 month-old healthy trees were inoculated via bud grafting using ‘*Ca*. L. asiaticus’-positive materials and maintained under the same conditions described above. To prepare phloem sap, five healthy (*Ct* > 37) and five ‘*Ca*. L. asiaticus’-infected (*Ct* < 25) trees were used to collect phloem sap using centrifugation of the separated bark tissues^57^, then kept at −20 °C for bioassays or further analysis.

### Insect materials, rearing conditions, and haemolymph collection

*Diaphorina citri* colonies were continuously reared at our laboratory at the CREC-IFAS-UF, Lake Alfred, FL, USA. Healthy psyllids were maintained on the ‛*Ca*. L. asiaticus’-free ‘Valencia’ sweet orange trees, which were pruned regularly to stimulate the growth of new flushes for optimal oviposition. For ‘*Ca*. L. asiaticus’-infected colonies, *D. citri* nymphs from the 4^th^ and 5^th^ instars were collected from the healthy (uninfected) laboratory population, then reared on ‘*Ca*. L. asiaticus’-infected ‘Valencia’ sweet orange trees and maintained under the same conditions as described above. For larvae of *G. mellonella* (greater wax moth), freshly molted (7^th^) instar larvae were obtained in sawdust from Gimminy Crickets & Worms (Surmen Legacy LLC, Little Ferry, NJ, USA, via Amazon). On the other hand, newly emerged honeybee (*Apis mellifera*) adults were kindly provided by Dr. Lukasz Stelinski, Entomology and Nematology Department - University of Florida^58^. The collection of *D. citri* haemolymph was carried out as described in our previous study^32^. Likewise, hemocyte-free haemolymph of *G. mellonella* was collected as described in our previous study^31^. The haemolymph of the honeybee was collected using the antennae method as previously described by ref. ^59^. All collected hemolymphs were used immediately for bioassay or stored at −20 °C until further analysis.

### Non-targeted metabolomics of haemolymph

For untargeted metabolomic analyses of haemolymph, three different derivatization reagents were used, including *N*-Methyl-*N*-(trimethylsilyl)trifluoroacetamide (MSTFA, also known as trimethylsilyl [TMS])^31,32^, methyl chloroformate (MCF)^31,32,57^, and boron trifluoride (BF₃)^60^, to ensure a comprehensive metabolic profile of haemolymph using GC-MS. Derivatized haemolymph samples/standards were injected into a GC-MS system model Clarus 680 (Perkin Elmer, Waltham, MA, USA) fitted with Elite-5MS capillary column (30 m × 0.25 mm I.D. × 0.25 µm film thickness; Perkin Elmer, Waltham, MA, USA) running in the full scan mode. Helium was used as the carrier gas at a flow rate of 1 mL per minute. The GC thermal program, MS ion identification, data acquisition, and chromatogram analysis were carried out as described in our previous studies based on the targeted metabolites and derivatization reagents of MCF^31,32,57^, MSTFA^31,32^, and BF₃^60^. The injector and the detector temperatures were set at 220 °C and 280 °C, respectively. All detected metabolites were initially identified by comparing their mass spectra with spectra from the published literature^31,32,57,60^. Then, the identification was confirmed using library entries and authentic reference standards.

### Energy metabolism and nucleotide analysis by HPLC

Nucleotides and sugar-nucleotides were extracted from the haemolymph of *G. mellonella* using perchloric acid as previously described^61^ and slightly modified by ref. ^30^. Briefly, 100 *μ*L of *G. mellonella* hemocyte-free haemolymph was extracted with 100 *μ*L of cold 5% perchloric acid, neutralized with 10 *μ*L of 10 N potassium hydroxide (KOH), ultra-filtrated through a 10 K MWCO membrane (10,000 kDa; Millipore, Bedford, MA), and then stored at − 20 °C until HPLC analysis. For HPLC analysis, 1 *μ*L of the haemolymph nucleotides extract was injected into an Agilent 1200 Series High-Performance Liquid Chromatography (HPLC; Agilent Technologies, Santa Clara, CA, USA), coupled with a diode array detector (DAD) and a Dionex™ CarboPac™ PA100 Column (Dionex, Sunnyvale, CA, USA) that kept at 25 °C. The mobile phase composition and other analytical conditions were exactly as described in our previous study^30^.

### Survival assay of ‘*Ca*. L. asiaticus’-infected Galleria

To test the viability of ‘*Ca*. L. asiaticus’ with infected *G. mellonella* larvae, 25 freshly-molted ultimate (7^th^) instar larvae (~0.2–0.3 g) were inoculated with ‘*Ca*. L. asiaticus’-infected phloem sap (*Ct* < 25) or ‘*Ca*. L. asiaticus’-infected *D. citri* haemolymph (*Ct* < 25). Phosphate-buffered saline (0.1 M, pH 7.4) containing 0.1% ascorbic acid (w/v) as an antioxidant (hereinafter referred to as PBS), ‘*Ca*. L. asiaticus’-free phloem sap (*Ct* > 37), and ‘*Ca*. L. asiaticus’-free haemolymph from *D. citri* (*Ct* > 37) were used as controls. PBS was used to dilute the haemolymph and phloem sap (ratio of 1:3 phloem sap/haemolymph to buffer, respectively). Briefly, *G. mellonella* larvae were initially surface-sterilized using 70% ethanol and injected with 10 µL of diluted *D. citri* haemolymph or ‘Valencia’ sweet orange phloem sap in PBS and ascorbic acid into the anal proleg at the end of the abdomen using a 10 µL Hamilton Microliter Syringe (Hamilton Company, Reno, NV, USA) coupled with Harvard Apparatus (Volume: 00.30 ml, 01.50 ml/min, target 0.30 ml; Harvard Bioscience, Holliston, MA, USA). Inoculated larvae were placed in Petri dishes padded with wet filter paper (5 larvae per plate) and maintained under controlled laboratory conditions at 27 ± 2°C, with 70 ± 5% RH, and an 8D:16 L photoperiod.

Survival of inoculated larvae was monitored every 24 h post-inoculation (hpi) until all larvae emerged as adults or died (100% death). *G. mellonella* larvae found on their sides or back, unable to move when prodded with a camel hairbrush, or completely melanized were considered dead. Survivorship and its associated lifespan (days) were analyzed using Kaplan-Meier survival curves along with log-rank tests. Larvae were sampled at 48 hpi to quantify the bacterial titer of ‘*Ca*. L. asiaticus’ using qPCR.

### Quantification of ‘*Ca*. L. asiaticus’ bacterial titer using qPCR

Bacterial titer of ‘*Ca*. L. asiaticus’ within inoculated *G. mellonella* larvae was quantified using quantitative real-time PCR (qPCR) at 0, 24, 48, 72, and 96 h post-inoculation (hpi) as described in previous studies^62–65^ and expressed as cycle threshold (*Ct*) values. Initially, *G. mellonella* (7^th^ instar larvae) were inoculated with ‘*Ca*. L. asiaticus’-infected phloem sap (*Ct* < 25) or ‘*Ca*. L. asiaticus’-infected *D. citri* haemolymph (*Ct* < 25) as described above. PBS, ‘*Ca*. L. asiaticus’-free phloem sap (*Ct* > 37), and ‘*Ca*. L. asiaticus’-free haemolymph from *D. citri* (*Ct* > 37) were used as negative controls. Briefly, whole larvae (five biological replicates, five larvae per replicate) were flash-frozen in liquid nitrogen and homogenized using a TissueLyser II (QIAGEN Sciences Inc., Germantown, MD, USA) at 30 Hz for five min with a sterile metal bead. After homogenization, DNA was extracted using a potassium acetate lysis method using a manual lysis/precipitation protocol adapted in our laboratory, then precipitated with ice-cold isopropanol, washed in 70% ethanol, air-dried, and resuspended in nuclease-free water^62,63^. DNA was purified and quantified using a NanoDrop 2000 spectrophotometer (Thermo Fisher Scientific, Waltham, MA, USA), and its concentration was adjusted to 100 ng μL⁻¹.

Purified DNA was used for qPCR amplification using a 16S rDNA-based TaqMan primer (HLBasf and HLBr) and probe (HLBp) sets specific to *Ca*. Liberobacter spp. as previously described by Li et al.^66^. with TaqMan PCR Master Mix on an ABI 7500 Real-Time PCR System (Applied Biosystems, Foster City, CA, USA). Due to the unculturable nature of ‘*Ca*. L. asiaticus’ in artificial media, as well as because of its low concentration and uneven distribution within its host plants and vector psyllids, a standard curve using purified genomic DNA was not possible. However, the used assay was previously reported to be very sensitive^66^ and did not show any cross-reactivity with other bacterial pathogens or endophytes commonly resident in citrus plants. Each run included Mock (PBS-treated), ‘*Ca*. L. asiaticus’-free tissue (citrus phloem sap and psyllid haemolymph) as negative controls, as well as ‘*Ca*. L. asiaticus’-positive citrus phloem sap and psyllid haemolymph. A *Ct* threshold of 32 was chosen based on reproducible detection in technical replicates and clear separation from background amplification in negative controls. Bacterial abundance was expressed as *Ct* values, which negatively reflect pathogen load^62–65^.

### Statistics and reproducibility

Throughout this study, all experiments were laid out using a completely randomized design (CRD) with five biological replicates and two technical replicates per treatment (*n* = 5), unless otherwise stated. For both the survival assay and bacterial titer quantification of ‘*Ca*. L. asiaticus’-infected Galleria, five biological replicates (each replicate contains five freshly-molted ultimate 7^th^ instar larvae) were used. The whole experiment was repeated twice. For non-targeted metabolomics of haemolymph (MSTFA, MCF, and BF_3_), as well as energy metabolism and nucleotide analysis by HPLC, the experiments were conducted using three independent biological replicates (each replicate contains five larvae), and measured in duplicate (two technical replicates). Technical replicates were excluded from the statistical analysis to prevent pseudo-replication. All data were tested for normality. The student *t*-test was used to compare the means of two groups (*D. citri* vs. *G. mellonella*), whereas analysis of variance (ANOVA) was used to compare the means of three or more groups, followed by the Tukey-Kramer honestly significant difference (HSD) test for post-hoc pairwise comparisons (*p* ≤ 0.05). False discovery rate (FDR) adjustment of *p*-values was conducted using the Benjamini-Hochberg False Discovery Rate (FDR) correction (*q* ≤ 0.05). *p*-values adjustment applied only to valid statistical tests. Comparisons that could not be made due to missing treatment groups were excluded from the FDR adjustment. Hierarchical cluster analysis (HCA) was performed using the raw cycle threshold (*Ct*) data matrices for the five treatments studied over five time-points (0, 24, 48, 72, and 96 hpi), and the multivariate *Ct* similarities were presented as a heat map. Survival analysis was conducted using the Kaplan–Meier method^67,68^ to describe the survival probability of *G. mellonella larvae* after inoculation with ‘*Ca*. L. asiaticus’. Kaplan-Meier-associated *p-*values and Chi^2^ (*χ*^*2*^) of log-rank and Wilcoxon tests were also calculated and presented within the survival plots for statistical comparisons between treatments.

### Reporting summary

Further information on research design is available in the Nature Portfolio Reporting Summary linked to this article.

## Supplementary information

Description of Additional Supplementary Files

Supplementary Data 1-9

Reporting Summary

## Data Availability

All data supporting the findings of this study are available within the paper and its Supplementary Information. All raw numerical data underlying the generated graphs/charts are provided in Supplementary Tables 1–9.
