# Supplementary material for: Galleria mellonella possesses the essential nutritional needs to host the fastidious Huanglongbing bacterial pathogen ‘Candidatus Liberibacter asiaticus’
Source: Commun Biol. 2025 Sep 30;8:1398. doi: 10.1038/s42003-025-08802-5 (PMC12484571; doi:10.1038/s42003-025-08802-5)
Supplement: Supplementary file 1 — Description of Additional Supplementary Files [file 42003_2025_8802_MOESM1_ESM.docx]

# **Description of Additional Supplementary Files**

**File name:** Supplementary Data 1.

**Description:** Raw numerical data of the cycle threshold (Ct) of real-time qPCR for the detection of ‘Ca. L. asiaticus’ bacterial titer within *G. mellonella* larvae exposed to infection with ‘Ca. L. asiaticus’ via C. sinensis phloem sap or D. citri haemolymph over 96 hpi (*n*=5). Data was used to generate fig. 1b and 1c.

**File name:** Supplementary Data 2.

**Description:** Raw numerical data of Kaplan-Meier analysis of survival probability of *G. mellonella* larvae exposed to infection with ‘Ca. L. asiaticus’ via C. sinensis phloem sap or D. citri haemolymph over four days post-inoculation (dpi) (*n*=25). Data was used to generate fig. 1d.

**File name:** Supplementary Data 3.

**Description:** Raw numerical data of lifespans associated with the cumulative survival of *G. mellonella* larvae with different ‘Ca. L. asiaticus’ infection (*n*=5). Data was used to generate fig. 1e.

**File name:** Supplementary Data 4.

**Description:** Raw numerical data of haemolymph chemical composition of Asian citrus psyllid (*Diaphorina citri*), greater wax moth (*Galleria mellonella*), and honeybee (*Apis mellifera*) after TMS derivatization using GC-MS (*n*=3). Data was used to generate fig. 3 and Table 1.

**File name:** Supplementary Data 5.

**Description:** Raw numerical data of Haemolymph chemical composition of Asian citrus psyllid (*Diaphorina citri*) and greater wax moth (*Galleria mellonella*), after derivatization with methyl chloroformate (MCF) using GC-MS (*n*=3). Data was used to generate Table 2.

**File name:** Supplementary Data 6.

**Description:** Raw numerical data of Haemolymph chemical composition of Asian citrus psyllid (*Diaphorina citri*) and greater wax moth (*Galleria mellonella*), after derivatization with methyl chloroformate (MCF) using GC-MS (*n*=3). Data was used to generate Fig. 4b.

**File name:** Supplementary Data 7.

**Description:** Raw numerical data of the percentage of different nucleotides and sugar-nucleotides and their energetic groups detected in haemolymph of *G. mellonella*. Data was used to generate Fig. 5d.

**File name:** Supplementary Data 8.

**Description:** Raw numerical data of AMP: ATP ratio, ADP: ATP ratio, and adenylate energy charge (AEC), respectively, of *D. citri* (*n*=3) and *G. mellonella* (*n*=6). Data was used to generate Fig. 5e, 5f, and 5g.

**File name:** Supplementary Data 9.

**Description:** Raw numerical data of nucleotides and sugar-nucleotides detected in the haemolymph of Asian citrus psyllid (*Diaphorina citri*), and greater wax moth (*Galleria mellonella*), using HPLC. Data was used to generate Fig. 6d.
