# Supplementary material for: Galleria mellonella possesses the essential nutritional needs to host the fastidious Huanglongbing bacterial pathogen ‘Candidatus Liberibacter asiaticus’
Source: Commun Biol. 2025 Sep 30;8:1398. doi: 10.1038/s42003-025-08802-5 (PMC12484571; doi:10.1038/s42003-025-08802-5)
Supplement: Supplementary file 3 — Reporting Summary [file 42003_2025_8802_MOESM3_ESM.pdf]

nature portfolio

Corresponding author(s): Dr. Nabil Killiny
E-mail: nabilkilliny@ufl.edu
Phone: 863 956 8833
Fax: 863 956 4631
Manuscript COMMSBIO-25-3339A

Last updated by author(s): Aug 15, 2025

Reporting Summary

Nature Portfolio wishes to improve the reproducibility of the work that we publish. This form provides structure for consistency and transparency in reporting. For further information on Nature Portfolio policies, see our Editorial Policies and the Editorial Policy Checklist.

Statistics

For all statistical analyses, confirm that the following items are present in the figure legend, table legend, main text, or Methods section.

n/a	Confirmed	
☐	☒	The exact sample size (*n*) for each experimental group/condition, given as a discrete number and unit of measurement
☐	☒	A statement on whether measurements were taken from distinct samples or whether the same sample was measured repeatedly
☐	☒	The statistical test(s) used AND whether they are one- or two-sided<br>*Only common tests should be described solely by name; describe more complex techniques in the Methods section.*
☐	☒	A description of all covariates tested
☐	☒	A description of any assumptions or corrections, such as tests of normality and adjustment for multiple comparisons
☐	☒	A full description of the statistical parameters including central tendency (e.g. means) or other basic estimates (e.g. regression coefficient) AND variation (e.g. standard deviation) or associated estimates of uncertainty (e.g. confidence intervals)
☐	☒	For null hypothesis testing, the test statistic (e.g. *F*, *t*, *r*) with confidence intervals, effect sizes, degrees of freedom and *P* value noted<br>*Give P values as exact values whenever suitable.*
☒	☐	For Bayesian analysis, information on the choice of priors and Markov chain Monte Carlo settings
☐	☒	For hierarchical and complex designs, identification of the appropriate level for tests and full reporting of outcomes
☒	☐	Estimates of effect sizes (e.g. Cohen's *d*, Pearson's *r*), indicating how they were calculated

Our web collection on statistics for biologists contains articles on many of the points above.

Software and code

Policy information about availability of computer code

Data collection	NA
Data analysis	All statistical analyses were performed using JMP Student Edition 18.2.1 (JMP Statistical Discovery LLC, Cary, NC, USA)

For manuscripts utilizing custom algorithms or software that are central to the research but not yet described in published literature, software must be made available to editors and reviewers. We strongly encourage code deposition in a community repository (e.g. GitHub). See the Nature Portfolio guidelines for submitting code & software for further information.

Data

Policy information about availability of data

All manuscripts must include a data availability statement. This statement should provide the following information, where applicable:

- Accession codes, unique identifiers, or web links for publicly available datasets
- A description of any restrictions on data availability
- For clinical datasets or third party data, please ensure that the statement adheres to our policy

All data supporting the findings of this study are available within the paper and its Supplementary Information. All raw numerical data underlying the generated graphs/charts are provided in Supplementary Data 1-9.

Research involving human participants, their data, or biological material

Policy information about studies with human participants or human data. See also policy information about sex, gender (identity/presentation), and sexual orientation and race, ethnicity and racism.

Reporting on sex and gender	Not applicable
Reporting on race, ethnicity, or other socially relevant groupings	Not applicable
Population characteristics	Not applicable
Recruitment	Not applicable
Ethics oversight	Not applicable

Note that full information on the approval of the study protocol must also be provided in the manuscript.

Field-specific reporting

Please select the one below that is the best fit for your research. If you are not sure, read the appropriate sections before making your selection.

☒ Life sciences ☐ Behavioural & social sciences ☐ Ecological, evolutionary & environmental sciences

For a reference copy of the document with all sections, see nature.com/documents/nr-reporting-summary-flat.pdf

Life sciences study design

All studies must disclose on these points even when the disclosure is negative.

Sample size	For both survival assay and bacterial titer quantification of 'Ca. L. asiaticus'-infected Galleria, five biological replicates (each replicate contains five freshly-molted ultimate 7th instar larvae) were used. The whole experiment was repeated twice. For non-targeted metabolomics of haemolymph (MSTFA, MCF, and BF3), as well as energy metabolism and nucleotide analysis by HPLC, the experiments were conducted using three independent biological replicates (each replicate contains five larvae), and measured in duplicate (two technical replicates).
Data exclusions	Technical replicates were excluded from the statistical analysis to prevent pseudo-replication.
Replication	All samples were measured in duplicate (two technical replicates). Technical replicates were excluded from the statistical analysis to prevent pseudo-replication.
Randomization	Throughout this study, all experiments were laid out using a completely randomized design (CRD)
Blinding	Not applicable

Reporting for specific materials, systems and methods

We require information from authors about some types of materials, experimental systems and methods used in many studies. Here, indicate whether each material, system or method listed is relevant to your study. If you are not sure if a list item applies to your research, read the appropriate section before selecting a response.

Materials & experimental systems

n/a	Involved in the study	
☒	☐	Antibodies
☒	☐	Eukaryotic cell lines
☒	☐	Palaeontology and archaeology
☐	☒	Animals and other organisms
☒	☐	Clinical data
☒	☐	Dual use research of concern
☐	☒	Plants

Methods

n/a	Involved in the study	
☒	☐	ChIP-seq
☒	☐	Flow cytometry
☒	☐	MRI-based neuroimaging

Animals and other research organisms

Policy information about studies involving animals; ARRIVE guidelines recommended for reporting animal research, and Sex and Gender in Research

Laboratory animals	For Diaphorina citri colonies, nymphs from the 4th and 5th instars were continuously reared at our laboratory at the CREC-IFAS-UF, Lake Alfred, FL, USA.<br>For larvae of G. mellonella (greater wax moth), freshly molted (7th) instar larvae were obtained in sawdust from Gimminy Crickets & Worms (Surmen Legacy LLC, Little Ferry, NJ, USA, via Amazon)
Wild animals	Not applicable
Reporting on sex	Not applicable
Field-collected samples	Not applicable
Ethics oversight	Not applicable

Note that full information on the approval of the study protocol must also be provided in the manuscript.

Plants

Seed stocks	The most common HLB-susceptible cultivar, 'Valencia' sweet orange (Citrus sinensis), was used as an experimental plant throughout this study.
Novel plant genotypes	Not applicable
Authentication	Not applicable
